# Knowledge Levels and Educational Experiences among Dental Graduates in Saudi Arabia Regarding Child Abuse and Neglect: A National Study

**DOI:** 10.3390/children8090724

**Published:** 2021-08-25

**Authors:** Ayman M. Sulimany, Abdulaziz Alsamhan, Abdulrahman Awwad Alawwad, Mohammad Aqueel, Nawaf Alzaid, Omar A. Bawazir, Hebah M. Hamdan

**Affiliations:** 1 Department of Pediatric Dentistry and Orthodontics, College of Dentistry, King Saud University, Riyadh 11545, Saudi Arabia; obawazir@ksu.edu.sa; 2 Intern, College of Dentistry, King Saud University, Riyadh 11545, Saudi Arabia; azoozsamhan@gmail.com (A.A.); mohammadiaqueel@gmail.com (M.A.); nawaf-alzaid@live.com (N.A.); 3 General Dentist, Ministry of Health, Riyadh 11176, Saudi Arabia; abdulrahmanbinawaad@gmail.com; 4 Department of Periodontics and Community Dentistry, College of Dentistry, King Saud University, Riyadh 11545, Saudi Arabia; hhamdan1@ksu.edu.sa

**Keywords:** child maltreatment, child abuse, child neglect, dental education, Saudi Arabia, dentist

## Abstract

Background: Child abuse and neglect (CAN) is considered a serious problem worldwide. Dentists have a significant role in recognizing and reporting CAN cases. Aim: The aim of this study was to assess the CAN-related knowledge and educational experiences among Saudi dental graduates. Design: Self-administered questionnaires were distributed to dental graduates from all dental schools in Saudi Arabia (*n* = 1552). Bivariate and multivariate logistic regression analyses were performed to assess the associations between knowledge level and different predictors. Results: A total of 988 dental graduates completed the questionnaire. The majority of them were dissatisfied with the amount of education they had received in their school (56.4%). Around 60% of the participants had inadequate knowledge regarding CAN. Graduates from government schools who received dental education about CAN and female participants had significantly higher odds of having adequate knowledge scores than others (odds ratio = 2.0, 3.1, and 1.7, respectively). Only 39.5% of the participants felt confident in their ability to identify CAN cases, and only 9.7% knew how to report such cases. Conclusions: Graduate dental students have insufficient knowledge about CAN. More time should be dedicated to educating students about this important topic in dental curricula.

## 1. Introduction

The World Health Organization (WHO) defines child abuse and neglect (CAN) as: child abuse or maltreatment constitutes all forms of physical and/or emotional ill-treatment, sexual abuse, neglect or negligent treatment or commercial or other exploitation, resulting in actual or potential harm to the child’s health, survival, development or dignity in the context of a relationship of responsibility, trust or power [1].

CAN is a serious phenomenon that has long-term negative effects on children’s lives and their communities [2]. The effects of CAN may exhibit as inappropriate changes in a child’s physical, social, psychological, and cognitive health [3]. There might be physical injuries noticed immediately, but the impact of CAN can lead to a disturbed mental state, along with behavioral changes [3]. Children with a history of witnessing CAN are more prone to conditions such as malnutrition, vision disturbances, hypertension, diabetes mellitus, and cardiac problems [3], with high chances of several psychological problems such as stress, anxiety, the development of an inferiority complex, low productivity, a lack of social skills, and depression [3].

According to a WHO report published in 2020, every three out of four children aged two to four years suffer physical or psychological violence. One in every five women reports sexual abuse during their childhood, and one in every three men report the same [4]. In the United States, the Centers for Disease Control and Prevention (CDC) stated in 2021 that one in every seven children had faced CAN in the previous year. A previous report published by the CDC reported 1840 deaths of children due to CAN in the United States in 2019 [5]. In Saudi Arabia, the National Family Safety Program reported the registration of 616 CAN cases in 2011, a number almost eight times more than the number of cases reported in 2010 [6]. However, these numbers are likely to underestimate the actual number of CAN cases because they are merely based on hospital-based registries. Unfortunately, a substantial number of CAN cases go unreported, indicating the greater-than-apparent severity of the problem [7].

Dentists play a significant role in identifying and reporting cases of CAN since they mainly deal with the head and neck areas, which are reportedly the most commonly affected in cases of abuse [8,9]. Additionally, a dentist can be the first person that a victim comes in contact with after an incident [9]. Furthermore, dentists usually see patients and their parents regularly, allowing them to recognize any changes in behavior or signs of CAN [10]. Many studies from different parts of the world have demonstrated inadequate awareness and a lack of training among dental practitioners toward the identification and management of cases of CAN [10,11,12,13]. Moreover, although they are ethically and lawfully obligated to detect and report the cases of CAN to social services or law enforcement agencies, most dentists lack sufficient knowledge regarding the reporting process, letting many of the cases go unreported [12,14,15,16,17].

In Saudi Arabia, Al-Dabaan et al. investigated the knowledge and experience of the Saudi Dental Society members regarding CAN and found that only 21% of the participants knew about the child protection policies in the regions of their practice [15]. Likewise, Mogaddam et al. investigated knowledge about child physical abuse among dental interns, postgraduate pediatric dentistry residents, and dentists practicing in dental colleges of the Jeddah city, Saudi Arabia, and they reported inadequate knowledge about the identifying and reporting of suspected cases of child abuse among the participants [11]. 

As responsible health care providers, dentists should be able to identify and report any cases of CAN they encounter. Dental education can aid in increasing awareness of the identifying and reporting process among the upcoming dental practitioners, which can further help in reducing CAN. The inadequacy of CAN-related knowledge among dental practitioners may be attributable to the deficiency related to this topic in the dental curricula [13]. To the best of our knowledge, studies assessing undergraduate dental education in Saudi Arabia with regard to CAN are limited, with no available national data about the knowledge of CAN among graduating dentists. Therefore, the aim of this study was to assess the knowledge of recently graduated dental students in Saudi Arabia regarding CAN and to evaluate the extent of training provided to the students for this subject. The results of our study are expected to aid in planning interventions to improve the knowledge of CAN and confidence in dealing with CAN cases among dental graduates.

## 2. Materials and Methods

### 2.1. Study Population and Data Collection

This nationwide cross-sectional study was conducted during the academic year of 2018/2019. In Saudi Arabia, new dental graduates are required to complete one year of internship training after they graduate before they can proceed with their future careers. The target population of this study was new dental graduates who were expected to finish their internship training program in the summer of 2019. Out of the 27 dental schools in Saudi Arabia, 23 had dental intern graduates, resulting in a total of 1552 dental interns. Data were collected between February and April 2019. The questionnaires were either mailed or handed in person to the intern’s representative of each dental school to distribute it to dental graduates. To increase the response rate, three reminders were sent to the intern’s representative in two-week intervals. After that, questionnaires were collected from each school’s representatives. 

### 2.2. The Questionnaire

The self-administered questionnaire consisted of 23 questions that were developed based on previous studies [11,12,14,15,18]. The questionnaire was formulated in the form of multiple-choice questions in the English language and consists of three parts. The first part contains questions regarding background information, including the sex, age, and educational institution of the participants. The second part includes questions about the participants’ educational experience regarding CAN and their confidence in their ability to identify and report CAN cases. The third section was designed to test the participants’ knowledge of CAN. 

### 2.3. The Validity and Reliability of the Questionnaire

The content validity for the questionnaire was assessed by two pediatric dentists and two dental public health practitioners who are experts in the field of CAN. They were asked to assess the comprehensiveness, phrasing, and importance of each question in the questionnaire. Minor adjustments to the questionnaire were made based on the experts’ comments to improve the comprehensiveness of the questions. The face validity was assessed by distributing the questionnaire to a group of dental interns (*n* = 10) to evaluate the clarity of the questionnaire. Moreover, test–retest reliability was assessed by asking ten dental interns to fill out the questionnaire two times with one week between each completion. The results were assessed using kappa statistics, which resulted in an average *k* of 0.81, indicating good reproducibility and reliability.

### 2.4. Ethical Approval

The study was conducted according to the guidelines of the Declaration of Helsinki, and ethical approval was obtained from the institutional review board (IRB No. E-18-3417, approval date: 18 November 2018) and the College of Dentistry Research Center (CDRC No. IR0304) of King Saud University. 

### 2.5. Statistical Analysis

Frequencies and percentages were used to report the categorical variables corresponding to the background characteristics of the study sample in addition to their knowledge and educational background regarding CAN. Knowledge scores were calculated for participants based on their answers to 11 knowledge-related items of the questionnaire. A score of 60% was used as a cut-off point between adequate and inadequate knowledge levels which was set according to the regular passing score in dental schools in Saudi Arabia [19]. A multivariate logistic regression analysis was used to assess the associations between knowledge level and different independent variables. The level of significance was set at 0.05. Data were analyzed using SAS 9.4 software (SAS Institute, Inc., Cary, NC, USA).

## 3. Results

### 3.1. Sample Characteristics

A total of 1552 surveys were distributed in 23 dental educational institutes. A total of 988 dental graduates completed the questionnaire, resulting in a response rate of 63.7%. Around 55% of our samples were women, with a mean age of 24.9 years. 

### 3.2. Educational Experience

Table 1 illustrates the participants’ educational experience regarding CAN. The majority of the participants (56%) graduated from government schools. Around 27% of the participants did not receive any dental education regarding CAN in their undergraduate training. The majority of the participants were not satisfied with the amount of education they received in their school on the subject (56.4%). Additionally, the vast majority (68.9%) felt that more time should be dedicated to educating students on CAN within the undergraduate curriculum.

### 3.3. Knowledge Regarding CAN

**Figure 1:**
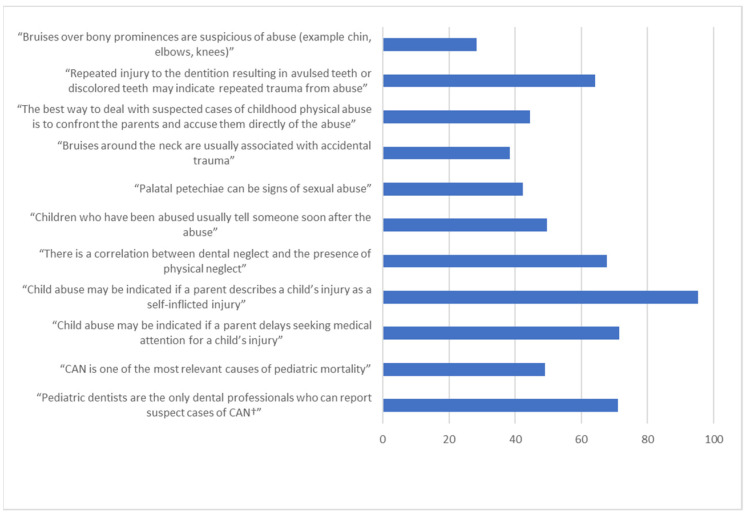
Percentages of the participants with correct answers to knowledge-based items in the questionnaire (*n* = 988). † Child abuse and neglect.

The mean knowledge score of our samples was 53.27 ± 21.06, with the majority of the participants (59.6%) had inadequate knowledge. Figure 1 presents the percentages of participants who provided correct answers to knowledge-based items in the questionnaire. Almost all the participants (95.3%) believed that a guardian reporting a child’s injuries as self-mutilation-based injuries is an indicator of CAN. Additionally, the majority of the participants (71.5%) agreed that there is an association between CAN and procrastination in seeking medical care for children’s injuries. Around 71% agreed that pediatric dentists are not the only dental professionals who can report suspect cases of CAN. The majority of the participants agreed that there is a correlation between physical and dental neglect, as well as that repeated dental injury can be a sign of CAN (67.8% and 65%, respectively). Additionally, 49.1% knew that CAN is the most prevalent cause of pediatric mortality. Half of the participants thought that victims of CAN tend to inform someone about the experience they faced soon after its occurrence, and more than half were not aware that palatal petechiae can be an indicator of sexual abuse. Less than 30% of the participants disagreed with the statement that bruises over the elbows and knees can be a sign of CAN. Only 44.4% of the participants agreed that confronting the parents and accusing them directly is not the most suitable approach for handling cases of CAN.

### 3.4. Determinants of Knowledge Level

Table 2 illustrates the participants’ knowledge scores in association with key variables of the study sample. The knowledge score was significantly associated with sex, type of school, and receiving dental education about CAN at dental school (0.002, <0.0001, and <0.0001, respectively). Around 45% of the female participants had adequate knowledge levels compared to 35% of the male participants. Only 31.2% of the participants from private dental schools had an adequate knowledge score compared to 47.6% of those from government schools. Around 48% of those who received dental education about CAN had adequate knowledge scores compared to 20.1% of those who did not. 

Table 3 presents the results of the multivariate logistic regression analysis. All the factors remained statistically significant after entering the model. Female participants had 1.7 times the odds of having adequate knowledge compared to male participants (confidence interval (CI): 1.3–2.3). Graduating from government schools increased the odds of having adequate knowledge by two times compared to those from private schools (CI: 1.5–2.7). Those who received dental education about CAN had 3.1 times the odds of having adequate knowledge compared to those who did not (CI: 2.2–4.4). 

### 3.5. Identifying and Reporting CAN

**Figure 2:**
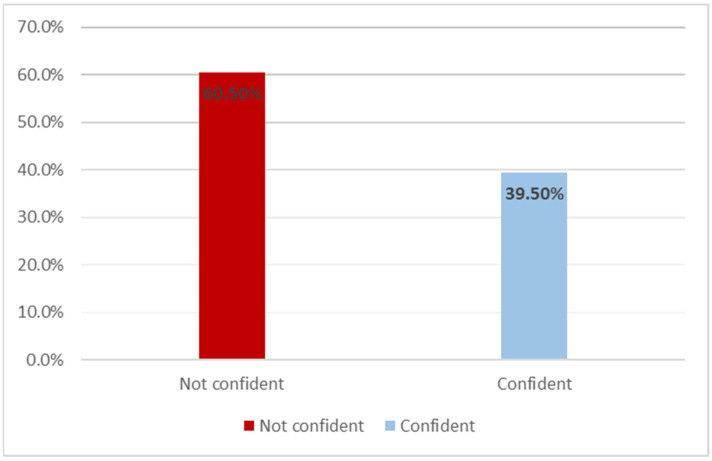
Distribution of the respondents with regard to their confidence in their ability to identify cases of child abuse and neglect (*n* = 988).

Only 39.5% and 39.8% of the participants felt confident in their ability to identify (Figure 2) and report cases of CAN, respectively. However, less than 10% of the participants knew where to report cases of CAN. 

## 4. Discussion

CAN is a major issue that affects a large number of children all over the world, and it may be an even larger problem than we realize due to underreporting. Dentists have an important role in suspecting and reporting CAN cases. This study was aimed to assess knowledge levels regarding CAN among new dental graduates in Saudi Arabia and to shed light on their educational experiences regarding this crucial topic during their undergraduate study years.

In our study, we found insufficient knowledge regarding CAN among dental graduates in Saudi Arabia. This is in agreement with the study conducted by Duman et al., in which they assessed knowledge regarding CAN among undergraduate students from 11 dental schools across the world [20]. Their study showed that dental students from Australia, Pakistan, Turkey, Jordan, Turkish Republic of Northern Cyprus, Poland, the Republic of South Africa, Nigeria, Cambodia, and the United States of America lack knowledge and experience in recognizing and reporting child abuse. 

In Saudi Arabia, Mogaddam et al. found similar results when they investigated knowledge regarding physical abuse among dental interns, postgraduate residents, and pediatric dentists in dental colleges of Jeddah city [11]. In contrast, Al-Dabaan et al. found that most of their participants had sufficient knowledge about CAN [15]. This may have been due to the difference in the study populations, as they targeted members of the Saudi Dental Society, which includes pediatric dentists who are experts in this field. Furthermore, the low response rate (1.6%) might indicate that only individuals who were interested in the research topic participated in their study. 

Our results indicate that graduates from government schools who received dental education about CAN and female participants had higher odds of having adequate knowledge scores than the other participants. Our findings are consistent with the results of the study conducted by Mogaddam et al., who found that the female sex and working in a government facility were significantly associated with higher knowledge scores [11]. Similarly, in a study aimed to assess the knowledge of and attitude toward CAN among dentists in Italy, Manea et al. found that women were more likely to correctly answer questions regarding CAN than men [21]. This may be attributed to the compassionate nature of women regarding children and their protection.

Dentists play a significant role in recognizing and reporting cases of CAN because the majority of the signs of abuse are usually seen in the areas they examine [8]. For this reason, dentists should feel confident in their ability to identify and report such cases. In our study, we found that less than 40% of the dental graduates were confident in their ability to identify CAN cases, and only 9.7% of them knew where to report such cases. These results are significantly lower than those of Mogaddam et al., who found that 63% of their participants knew where to report suspected cases of CAN [11]. This may have been related to their sample’s large proportion of postgraduate pediatric dentistry residents and pediatric dentists, who are expected to have more expertise in this field than our sample population. 

Despite the important role of education in improving knowledge regarding CAN, only 73% of the participants in our study stated that they received education about CAN during their undergraduate studies. This can be considered low compared to studies conducted in other countries. In a study conducted by Jordan et al., almost 80% of the Croatian senior dental students were found to have received training regarding CAN during their undergraduate training [10]. In a study targeting dental students at the University of Michigan, the United States, Thomas et al. found that all the study participants had learned about the topic in their senior year [14]. In Saudi Arabia, the law of protection from CAN is considered new compared to those of other countries because it did not become official until August 2013 [22]. This may explain the deficiency related to this topic in undergraduate dental curricula in Saudi Arabia, which further highlights the need to update the dental curricula to ensure that dental graduates have adequate knowledge regarding this important topic. 

The main limitation of our study is related to the self-reporting design of the study, which made it more prone to recall bias. Despite this limitation, to the best of our knowledge, this study is the first nationwide study to assess the knowledge and educational levels regarding CAN among dental graduates in Saudi Arabia. The large sample size with a relatively high response rate (63.7%) indicates a reduced risk of non-response bias, supporting the validity of the study. Our findings can be the foundation for establishing future interventions to improve graduates’ knowledge and confidence levels in identifying and reporting CAN cases. The intervention may include changing dental schools’ curricula by conducting awareness programs, seminars, and lectures; incorporating clinical workshop-based training; and initiating collaborations with different organizations to establish a multidisciplinary approach to handle suspected cases of CAN. Training should focus on increasing the student’s knowledge regarding the epidemiology, signs, and risk factors of CAN. Moreover, it should introduce Saudi Arabia’s child protection laws to dental students, highlight the importance of this crucial issue, and provide step by step standardized procedures for reporting suspected cases of CAN. Available evidence indicates training increases knowledge and changes attitudes [23], subsequently increasing the number of incidents reported [24]. Moreover, our results can be used as baseline data for evaluating the effectiveness of any possible future interventions that are aimed at improving graduates’ knowledge.

## 5. Conclusions

Our study demonstrates that there is insufficient knowledge regarding CAN among dental graduates in Saudi Arabia. Graduating from government schools, female sex, and receiving dental education about CAN were associated with higher knowledge levels on CAN; however, the majority of the participants did not feel confident in their ability to identify or report cases of CAN. Our findings indicate that dental curricula should be revised to ensure that dental graduates are confident in dealing with suspected cases they might encounter during their future careers. 

## Figures and Tables

**Table 1:** Participants’ educational experience regarding CAN † (*n* = 988).

	*n* (%)
Type of dental education	
Government	552 (55.9)
Private	436 (44.1)
Received dental education about CAN at dental school	
No	264 (26.7)
Yes	724 (73.3)
Satisfied with the amount of education received in dental school regarding CAN	
No	557 (56.4)
Yes	431 (43.6)
Felt that undergraduate curriculum should dedicate more time to educate about CAN	
No	152 (15.4)
Yes	681 (68.9)
I do not know	155 (15.7)

† Child abuse and neglect.

**Table 2:** Participants’ knowledge scores in association with key variables of the study sample (*n* = 988).

	Knowledge Scores		*p* Value
	Inadequate *n* (%)	Adequate *n* (%)	
Sex			0.002
Male	289 (64.9)	156 (35.1)	
Female	300 (55.2)	243 (44.8)	
Type of dental school			<0.0001
Government	289 (52.4)	263 (47.6)	
Private	300 (68.8)	136 (31.2)	
Received dental education about CAN † at dental school			<0.0001
No	211 (79.9)	53 (20.1)	
Yes	378 (52.2)	346 (47.8)	

† Child abuse and neglect.

**Table 3:** Results of the multivariate logistic regression analysis (*n* = 988).

	Odds Ratio	95% CI ‡
Sex		(1.3–2.3)
Male	Ref	
Female	1.7	
Type of dental school		(1.5–2.7)
Governmental	2.0	
Private	Ref	
Received dental education about CAN † at dental school		(2.2–4.4)
No	Ref	
Yes	3.1	

† Child abuse and neglect, ‡ 95% confidence interval.

## Data Availability

The data presented in this study are available on request from the corresponding author. The data are not publicly available.
